# Use of the *Value of Respite Model* for practical program evaluation

**DOI:** 10.3389/frhs.2026.1568798

**Published:** 2026-05-22

**Authors:** Susan Janko Summers

**Affiliations:** ARCH National Respite Network and Resource Center, United States

**Keywords:** practice evaluation, program evaluation, respite, respite research, short break

## Abstract

Two novel documents, *Measuring the Value of Respite* and a corresponding document, *Recommended Common Data Elements for Respite Research*, were developed by ARCH National Respite Network and Resource Center and the Committee for the Advancement of Respite Research to provide a practical framework for respite research and to identify data elements and measures that align with the *Value of Respite Model*. Concurrent with the development of the two documents, four respite (short break) programs in the U.S used drafts of both *The Value of Respite Model* and *Recommended Common Data Elements for Respite Research* to guide and inform them in an 18-month program evaluation grant project. As part of the project, ARCH conducted semi-structured interviews with the four evaluation project grantees to assess their understanding of evaluation concepts and design, as well as the perceived utility of the *Model* and *Recommended Common Data Elements* for program evaluation. Three of the four programs also produced formal data summaries based on their evaluations. Together, the interviews and data summaries shed light on whether, how, and under what conditions the *Value of Respite Model* and *Recommended Common Data Elements for Respite Research* were useful to respite programs seeking to conduct practical program evaluation and document the value of the respite services they provided to family caregivers and care recipients.

## Introduction: defining respite for research and evaluation

The overriding mission of the ARCH National Respite Network and Resource Center is to assist and promote the development of quality respite, or short break, programs in the United States. During the past decade, ARCH's work in promoting quality respite services led to a particular focus on research, evaluation, and respite best practices initiatives that could inform a collective understanding of what quality respite services mean.

Respite is generally defined by ARCH as “planned or emergency care provided to a child or adult with special needs in order to provide temporary relief to family caregivers” (https://archrespite.org). While this definition may make respite as a concept or service understandable to a range of caregivers or service providers, it doesn't necessarily serve the purposes of research or evaluation.

In 2014, ARCH established an Expert Panel on Respite Research composed of academics, researchers, service providers, advocates, policy makers, and administrators representing a range of age groups, disabilities, and professional disciplines. They were charged with: exploring the status of respite research; proposing strategies to overcome barriers to research; and developing a plan to encourage rigorous research in key areas that would translate to meaningful strategies and approaches to care.

Early in their deliberations, the Expert Panel noted the number of varying definitions used by researchers and how those definitions often confounded the interpretation of findings and comparisons across studies. (In fact, five different major federal programs in the United States at the time—aging, veterans, disabilities, child abuse populations, and home and community-based care services—, each defined respite differently for funding and policy purposes.) The Expert Panel cited the lack of a common, concise, working definition of respite as a barrier to respite research, and as their initial task, the Panel developed a definition that would lend itself to research and allow conversations across studies, professional disciplines, and programs. Their definition follows:

Respite is planned or emergency services that provide a caregiver of a child or adult with a special need some time away from caregiver responsibilities for that child or adult, and which result in some measurable improvement in the well-being of the caregiver, care receiver, and/or family system (4).

This definition emphasizes the importance of documenting outcomes related to caregiver, care recipient, or family “well-being” as a result of their using respite services.

To continue the Expert Panel's original work and generate recommendations for future respite research, ARCH established the Committee for the Advancement of Respite Research (CARR). The CARR proposed the *Value of Respite Model* to guide researchers in identifying, developing, and expanding evidence-based and -informed respite (or short break) services that improve caregiver outcomes (1). The *Value of Respite Model* is a multi-dimensional framework that incorporates theory and design from the Human or Social-Ecological Model (5, 6), the Life Course Framework (7), and Individual and Family Self-Management Theory (8).

**Figure 1:**
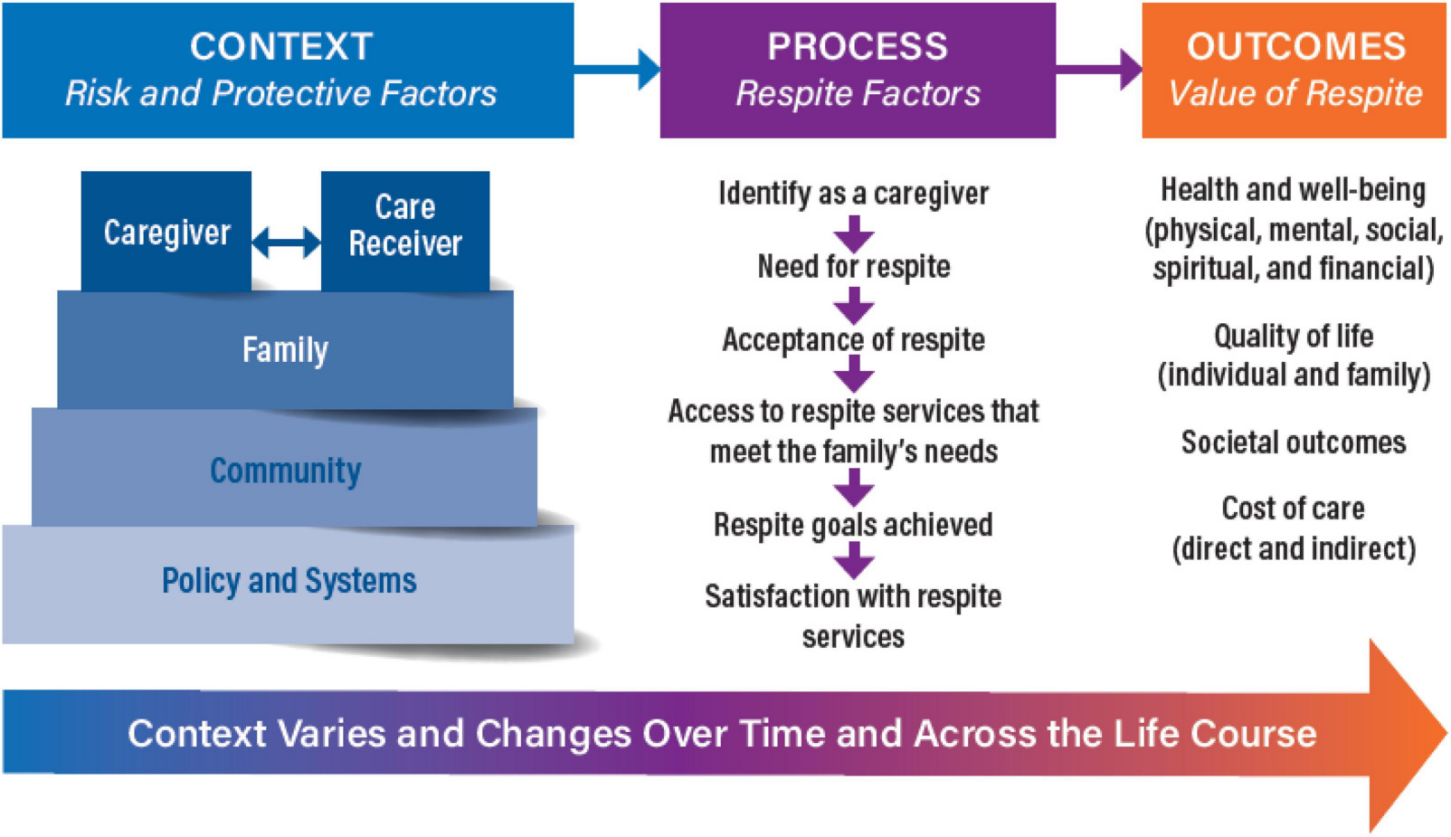
The Value of Respite Model (3).

The CARR subsequently developed *Common Data Elements*, precisely defined measurement questions and response options that align with the *Value of Respite Model* core concepts of measuring contexts, service processes, and outcomes. The CARR proposes that these *Common Data Elements* can guide data collection across studies and populations “allowing meaningful comparison of data across and between studies, between respite models, between study populations across age, disability, culture, and caregiving circumstances, or over time” (2) Please see Figure 1—The Value of Respite Model.

Concurrent with the development of *Common Data Elements*, four respite programs in the U.S. were awarded mini-grants by ARCH to support them in conducting individual program evaluations over a period of 18 months. ARCH's technical assistance to grantees was based in large part on using the *Value of Respite Model* and *Common Data Elements* to plan and conduct their evaluations. An unstated goal of the project was that grantees would find their evaluation plans sufficiently useful to sustain implementation as part of ongoing program development and evaluation after project funding ended. This goal was achieved by three of the four programs.

## Materials and methods

The four respite programs were selected via a competitive grant process from a pool of invited respite programs that had previously been recognized by the ARCH National Respite Network and Resource Center as Innovative & Exemplary Respite Services. ARCH launched an Innovative & Exemplary Respite Initiative in 2019 in order to establish a registry of respite programs and services in the U.S. that might foster our collective understanding of what respite “best practices” mean. Programs recognized as Innovative & Exemplary met all of the following seven stringent criteria: (1) an evidence-based or -informed approach; (2) a written program plan with goals and objectives; (3) a program service manual or guide; (4) person- and family-centered services; (5) a professional development plan; (6) an evaluation or data collection plan; and (7) a sustainability plan or activities (https://archrespite.org/provider-resources/innovative-and-exemplary-respite-services/).

It is notable that although all programs recognized by ARCH as Innovative & Exemplary met the seven criteria, relatively few were robust in meeting program evaluation criteria. While all recognized programs engaged in some evaluation activities, most relied primarily on caregiver satisfaction, in addition to basic demographic data, to assess the merit of their respite services. The majority lacked a comprehensive, coherent, written evaluation plan that included process and outcome measures directly linked to program goals and program activities. Although all programs collected information that guided decisions about services, program leaders often did not recognize that information as “data,” nor did they view the process of using that information to make informed decisions as practice development or performance evaluation. During in-depth interviews conducted as part of the application process, however, program leaders spoke in detail about how and why they developed and innovated services, and they shared their hunches and questions about how, whether, and in what ways their services worked for caregivers and their families.

### The evaluation project

We at ARCH reasoned that although respite program leaders were deeply knowledgeable and intellectually curious about their programs, they often lacked both practical experience and a useful framework and shared language to support in-house program evaluation or effective collaboration with external evaluators. Without adequate evaluation experience and useful tools, programs seemed unable to investigate and document whether and how respite produced measurable benefits for caregivers and families. The respite evaluation project was designed to provide an evaluation framework, language, and support in real-time, using the *Value of Respite Model* and *Common Data Elements* (Table 1). *Use of the Value of Respite Model to Measure Content, Process, and Outcomes*, summarizes data elements the four programs selected for their evaluations.

**Table 1:** Use of the value of respite model to measure content, process, and outcomes.

Context: Demographic, Caregiving, & Life Circumstance Data (personal, historical, and concomitant data that describe populations and may influence, mediate, or help with interpretation of outcomes)	Process: Respite Service Model Data* (independent and mediating variables, identified as part of a *Theory of Change*—the goals, methods and assumptions upon which service is based)	Outcome Data** (dependent variables resulting from or attributed in part to service model)
Caregiver(s) Care Recipient Family (immediate, extended) Neighborhood/Community	Evidence-Based and - Informed Models Dose Time Use Setting Individual/Shared Warm intake/onboard process Matching processes Coaching processes Communication processes Staff recruitment, training, supervision, support Organizational and social climate Peer-to-peer and community relationships Respite as a portal to complementary support services	Caregiver Wellness Physical Well-Being Emotional Well-Being Social Well-Being Positive Aspects of Caregiving Care Recipient Wellness Family Wellness Caregiver(s) Satisfaction Care Recipient Satisfaction Family Satisfaction Community Changes Societal/Policy Changes

* Respite Process requires both the documentation of service elements and measures of fidelity to model (how and how often services are delivered, and whether they are delivered as intended).

** Outcome data are typically measured pre- and post-service/intervention using psychometrically sound instruments that map onto stated program goals. Outcome data may also be measured at intervals or incrementally through goal attainment scaling that assesses individual progress toward goals, and may be collected in tandem with concomitant and mediating data to aide in understanding context that influences or explains progress toward outcomes.

In designing the evaluation project, ARCH project staff were also guided by an addendum to the Expert Panel's definition of respite included in the *Agenda to Advance Respite Research* (4). The addended text emphasizes the importance in research of: identifying who is expected to benefit from respite services; describing the personal characteristics of anticipated beneficiaries; describing service models in detail; and identifying measures that capture both expected outcomes and the context and process variables identified as likely to influence expected outcomes. The Expert Panel's statement captures the inherent complexity of respite or short break services.

Services provided may be informal, formal or specialized, and they may be provided to either, or both, the caregiver and the care receiver. It is acknowledged that respite takes on various forms; may be of short or extended duration; may occur one time, multiple times as needed by the caregiver, or be regularly scheduled; may include paid or voluntary services; may involve different types of providers and services of varying degrees of formality; may be provided in- home or at some other location (such as a center or camp); may involve or require direct staff with varying degrees of experience or training, or who possess various credentials; and may be designed to address different chronic or disabling conditions, types of dependency, age levels of dependent persons, and levels of dependency. To the degree that one or more of these variables are suspected of or intended to affect the desired outcomes for either caregivers or care receivers, they should be so acknowledged, measures should be identified, and the measures should be tracked throughout the research project for analysis and model testing (p. 14).

To support the four programs in designing evaluation plans that captured some of the inherent complexity of respite services, the respite program leaders met virtually monthly during an 18-month period for mini-lectures, individual reports on progress, and group discussions. In addition, each program met individually with ARCH staff multiple times throughout the project to develop evaluation plans, monitor and adapt data collection activities, and develop evaluation briefs.

ARCH asked programs to design evaluations that included the following six components:

1. **Statement of program goal or goals**. Program goals served as the foundation for outcome measurement and guided the content of respite “service maps.”
2. **Demographic and life circumstances data**. Demographic data corresponded to Context in the *Value of Respite Model*. Life circumstance data was also identified by some programs as important to collect. (For example, in crisis respite programs, challenging life circumstances are important for contemporaneous service planning, and they also provide context important for interpreting outcomes.)
3. **“Service map” of respite factors.** “Service maps” correspond to the *Process* component of the *Value of Respite Model*. Service maps document respite dose as well as other program activities believed to contribute to achievement of program goals. Following the evaluation project described here, and as part of the *Common Data Elements framework*, the CARR developed the *Respite Model Description Tool* (9). This tool should be a valuable resource for program evaluation in the future in capturing in checklist form key elements of respite services, including program content, characteristics, timing, and dose.
4. **Measurement processes and tools that align with goals, service activities, and outcomes and that document model fidelity**. When programs explicitly state program goals and specifically describe what their program is doing to achieve those goals, they are in effect stating their “theory of change” (10). A theory of change represents a working hypothesis of how and why specific services are expected to result in positive outcomes. In evaluation parlance, a theory of change reflects the proposition that: “If I arrange particular antecedents or create specific conditions, a desired outcome is more likely to occur.” It follows that evaluation measures should not only seek to capture anticipated outcomes, but should also specify the relevant antecedents and conditions (that is, the “service map”), and document the fidelity with which the planned antecedents or conditions that constitute respite service are implemented.
5. **Data triangulation across sources and methods.** Programs included three data sources in their evaluations: (1) caregiver and care receiver contexts, that is, demographic and caregiving and life circumstances (family and student contexts were added in two program evaluations); (2) “service maps” of respite process factors; and (3) outcomes (focusing on caregiver wellness consistent with the stated goals of each of the four programs). Programs were introduced to a field study approach (11) that includes quantitative data (enumeration and sampling) and qualitative data (observations and interviews) to aide them in data triangulation during data collection and interpretation.
6. **Outcomes.** Programs selected outcome measures (prior to the publication of the *Common Data Elements* but informed by their development) based upon the extent to which those measures aligned with their program goals.

In Table 2. Four Evaluation Project Programs, each of the four programs are briefly described in terms of their service model and population, goals and outcomes, evaluation methods, and data elements. Note that the *Value of Respite Model* accommodates the variety of persons served and service approaches used.

**Table 2:** Four evaluation project programs.

Program model	Goals/outcomes	Evaluation methods	Data elements
In-home respite supporting caregivers of adults with physical disabilities, dementia and memory conditions, chronic illness, and special medical needs.	Reduction in caregivers’ perceived stress and burden. Caregivers’ satisfaction with respite time-use and respite services.	Data collected at service intake. Two-month follow-up, and an annual evaluation on service anniversary. Follow-ups conducted via phone.	Demographic data Respite dose Service map University partner- designed, program-specific survey of caregiver satisfaction with respite time use and respite services. Zarit Scale of Caregiver Burden
In-home, community-based, and center-based respite from 2 h up to 10 days, for caregivers of persons with intellectual and/or physical disabilities from preschool age through the lifespan.	Reduction in caregivers’ perceived stress. Caregivers’ satisfaction with respite time-use and respite services.	Data collected at service intake and discharge. Daily monitoring and charting of care receiver well-being and goals. Regular periodic fidelity checks with families.	Demographic data Respite dose Service map Program-specific survey of caregiver satisfaction with respite services.
Center-based crisis respite care offered 24/7, 7 days weekly and 365 days yearly for caregivers of children birth through 12-years experiencing challenging life circumstances.	Relief of immediate stress experienced by caregivers and children during crises. Engender positive parenting and family relationships through ongoing education and support post-crises.	Data collected at service intake and discharge. Qualitative study conducted by university partner using surveys and interviews	Demographic data Respite dose Service map Qualitative surveys and individual interviews to discover caregiver perceptions of wellness, and whether and how caregivers believe respite supports and enhances wellness.
University-based, student provided pediatric respite for caregivers of children with special health care and developmental needs.	Reduction in caregiver stress. Increased caregiver self-efficacy. Increased student self-efficacy. Student mastery of core competencies.	Data collected at service intake and discharge. Student-family matching process. Regular periodic fidelity checks with families and students.	Demographic data Respite dose Service map University-designed, program specific survey Documented caregiver mentoring of students. Documented caregiver-to-caregiver education.

Despite monthly seminars and individual meetings during virtual “office hours”, about six months into the 18-month project, ARCH staff members (who were trained evaluators, and one had published and taught qualitative research in higher education settings) felt uncertain about how well programs understood evaluation concepts, and whether they were able apply those concepts in practice. The author (an educational ethnographer) scheduled time with each program's evaluation team to conduct semi-structured interviews. Interview questions were designed to help ARCH better understand how program evaluation team members conceptualized the *Value of Respite Model* and understood practical program evaluation techniques, and to identify how ARCH could best support each program during the final project months and not as a formal study of the *Value of Respite Model*.

Three initial questions were asked during the interviews.

1. *Describe the services you offer and how they benefit caregivers and others using them.* (This question was designed to capture the Respite Process identified in the *Value of Respite Model*, the program leader's Theory of Change, and program outcomes explicitly identified or implicitly valued by the program.)
2. *Talk about how you make sure things happen in your program as you have planned.* (This question was designed to capture program activities aimed at monitoring fidelity to program models.)
3. *What advice would you offer to other respite programs considering program evaluation?* (This question was designed to indirectly solicit reflections on challenges encountered in designing and conducting evaluations and possible solutions and supports.)

Embedded impromptu questions were asked to probe for clarity, to collect additional explanatory information, and to check the interviewer's understanding in real time.

Interviews lasted from 40 min to one hour. All interviews were conducted by the same interviewer. Two interviews were conducted with a single program evaluator from each of two programs. The remaining two interviews were conducted with program evaluation teams. One team included two persons. The second team included four persons who responded during the interview and another three persons (including student interns) who observed the interview. Interview transcripts provided a convenience sample and were not designed as part of a formal study of the *Value of Respite Model*.

Interviews were audio-recorded and transcribed verbatim. Each interview yielded approximately 4,000 words for a total of just over 16,000 words. Approximately 12 percent of spoken words are attributed to the interviewer and 88 percent of spoken words are attributed to those interviewed. The ratio of questions to answers are possibly an indicator of the depth of knowledge possessed by those interviewed, and how little prompting they needed to speak in depth about their programs. The interviewer was well known to program leaders, and interviewer-program relationships were collegial which likely also contributed to the ease programs felt answering open-ended questions and thinking aloud about their work.

An Inductive Thematic Analysis strategy (12) was used to discover patterns of thinking and meaning (or themes) derived from the data (rather than themes determined *a priori*) and related to the three broad interview questions (13): (1) respite service models and processes and the programs' theories of change; (2) whether and how programs established fidelity to their service models and processes; and 3) insights about program evaluation itself. Interpretations of the data relative to the *Value of Respite Model*, consistent with thematic analysis, are presented in the following along with primary data. All persons quoted in this article provided written permission for inclusion of their quotes. All those quoted also were given a draft of the article so they could review their quotes in context along with the author's interpretations.

Please note that because the data shared here were collected as part of an ongoing evaluation study rather than a planned research study, the author did not keep a reflexive journal as part of data collection and analysis. However, my own experiences as a caregiver changed significantly during the 18-month evaluation period. While analyzing data, I realized that I was not receiving the care that the four programs described as priorities—listening to, considering, and honoring caregiver and family perspectives in real time. My changed circumstances gave me a deeper, lived understanding of these priorities, making them feel more important and urgent than I might have appreciated otherwise.

## Results

### The value of respite model—respite processes and outcomes: the primacy of relationships

**Question 1.** Describe the services you offer and how they benefit caregivers and others using them.

The overriding purpose of interviews was to allow ARCH to informally assess each program's progress in designing and conducting an evaluation that would result in a data summary that corresponded to the *Value of Respite Model* and *Common Data Elements*. We asked open-ended questions that, rather than soliciting answers to what we thought was most important about their evaluations, allowed those interviewed to answer in any way that made sense to them and, we hoped, would reflect their priorities.

Please note that all of the following excerpts were the first responses from programs to the first question we asked. We report this original source data in narrative form rather than excerpting individual quotes so that readers can hear program leaders as they “think aloud” about their work. Without exception, programs were unhesitating with their answers, and the content of responses was dense without the interviewer needing to prompt for additional information.

### Forming close, trusted relationships

Program One describes service delivery as beginning with the act of listening.

What I've seen when I go out for a visit, even when I go out for an hour on the first visit, caregivers know there's something out there for them, that they're not alone, that someone is listening to them. There's a lot of value just in that. And then when you have a person attached, a consistent person that they communicate with, six or seven times out of ten, those relationships become close, trusted relationships… (Caregivers) feel like they're not alone. Usually, the benefit is to one primary caregiver… (and for extended family) it's that peace of mind. Someone else is checking on mom and dad. It takes something off of them.

Notice that for Program One, forming “close, trusted relationships” is part of the respite service and constitutes a foundation for respite, and that primary caregivers are the first beneficiary of that service. Program One then specifically describes what the program does to establish a close, trusted relationship.

Being in someone's home, you want them to feel at ease… It's a lot about the listening. A lot of times, caregivers just have held it in, and you hear it all. So, it's a good time to listen, and let them know what resources are available. And I let them know that I’m part of the package, so if (they) have questions at all or need anything, give a call or email me, and I can at least point (them) in the right direction. I'm a resource for (them) on an ongoing basis… We immediately set the tone that “we are here to support you.” And we're genuine. We believe in the services we're providing. We believe in our program. So, it's real easy to sell it…We've established very open (communication). Call us, email us, text us, if you have an issue. Let us know how else we can support you. Use us as your primary caregiving resource. As you need other resources, we'll help you get them.

Program Two also describes services and relationships as beginning at the first point of contact.

I think from the get-go our intake process is really good. We've tweaked it over the years and it seems to be working better for families to go slow and to increase as we go. Part of our intake process is to have families come for four hours initially, and we don't bill for that as a transition in and observation to see how the first four hours goes. And then, if they're going to do extended stays, we have them do one overnight before they do an extended three-to-five days, or however long they're going to stay, just to make sure their loved one is comfortable being in the house. A lot of people are just really hesitant to use respite sometimes. They feel like they're not doing what they’re supposed to be doing, or they feel that guilt of ‘I can’t believe (I’m) taking that vacation after 20 years,' and feeling guilty about it. So, I think that building those relationships and nurturing them initially is so important. Because you have to build those trusting relationships in order for anyone to feel comfortable with care from someone other than the primary caregiver.

For Program Two, “building those relationships and nurturing them initially is so important.” Like Program One, Program Two describes awareness of caregiver perspectives from the outset, and specifically describes what the program does to “build those trusting relationships in order for anyone to feel comfortable with care from someone other than the primary caregiver.” Services are available from the start on a trial basis, but together programs and families “go slow” and “increase as we go,” allowing time to build trusting relationships.

Program Three also responds quickly to families in crisis, and recognizes that a trusting relationship will take time to grow.

It's really multi-faceted. The parents get help in the moment for whatever brought them to our door. They've got a safe place for their children while … they get that immediate break to deal with what they need to. But I think it goes deeper than that. The fact that our families get a level of support that they're not alone in this. I think that is huge. Our staff comes at it with a very non-judgmental perspective, which I don't think many of our families are used to. I think that has a profound impact on how they see themselves. How they see themselves…as worthy of being supported.

We really try to not turn away a new family because we know if we do, we lose access to them. They're not calling a second time. That means if we have to bring in extra staff, or if a supervisor needs to be on the floor, we're going to do that the first time they call so they get at least that first dose of connection with us.

For Program Three, offering respite to families experiencing difficult life circumstances, program staff need to “come at it with a very non-judgmental perspective.” The program believes this approach “has a profound impact on how they see themselves. How they see themselves…as worthy of being supported.” Again, we see that establishing a connection with caregivers is the priority during initial contacts with programs, and that programs are clear and intentional in what they need to do to assure that families receive “at least that first dose of connection with us.”

The key takeaway from program responses to the first question is this: the primary service that programs described wasn’t an actual service in a way we traditionally think, such as, a schedule or respite dose, or a service location or activity. Rather, programs described a way of designing and delivering respite services through their relationships with families.

### Reciprocal, mutually beneficial relationships

Program Four, a university-based respite program, held an inclusive view on relationships thinking of them in terms of benefits accruing to children, caregivers, and students.

We provide relief for the caregivers. The way we do that is our nursing students go and take care of children with disabilities within the community so that their caregivers can take a break. And that break looks very different for all caregivers. For some, they'll take a nap at home for two hours while the students care for their children. Some will go out and do errands. Some spend time with the other siblings in the family…And…it helps caregivers because they are able to find qualified caregivers. I think that's really big, especially when it comes to a child with a disability…

(And) it is mutually beneficial. We have heard from our caregivers, and we see it. I just read a care plan last night from a student who talked about this in response to the question: *What does the family hope to get out of this?* The (parent) hopes to be able to educate future nurses on how to care for individuals with disabilities or chronic health care issues…It really gives our caregivers empowerment. I really advise students to go in with the attitude that the parents and the caregivers are the experts on their child. We can read about it in a text book, and we certainly want them to prepare in advance of their visit, to read about the child's conditions. But the caregivers are the ones who are with the child and really are the experts. So, it gives (parents) the opportunity to educate…

For Program Four, benefits are believed to accrue to both caregivers receiving services and to students learning to provide services. The program describes the importance of students learning “soft skills”—empathy, communication, and joint problem solving—as they provide services and learn from parent caregivers.

We have an orientation every semester for the nursing students, … and I talk about the school of nursing technical standards, and quote “soft skills.” And it can sound kind of demeaning calling them soft skills, but they’re super, super important. I think in nursing the hard skills are seen as more important, like getting blood pressure and drawing blood. But (they) leave out communication in working with the families. …Working on the communication aspect, I emphasize empathy in general. In-home care is a very unique experience for health care professionals, and it’s a very different environment than meeting in an acute setting where they see the patient for five minutes then review paper medical records, and don't get to see what's going on in the home, how the siblings interact, the fact that the parent can't go out and get groceries, and all these different things. Empathy. Problem solving.

Program Four regards caregivers as the primary beneficiaries of respite and holds that for caregivers to benefit, respite must be tailored to family circumstances. The program also views caregivers as experts and trains students to solicit and hontor caregivers' expertise—an approach believed to benefit both caregivers and students. Program Four illustrates how relationships between caregivers and respite programs create conditions necessary for knowledge sharing, which can result in services that better match caregiver needs while simultaneously strengthening individual respite services and the program as a whole. By explicitly valuing caregivers' knowledge, expertise, and wisdom, these relationships also foster caregiver self-efficacy and self-advocacy.

In designing the evaluation project, ARCH did not anticipate that programs would converge around the primacy of relationships and the role programs play in forming and supporting them. We had expected to hear more about outcomes traditionally emphasized in respite literature—ways to measure caregiver stress or burden, for example—outcome measures that ARCH promoted during monthly evaluation meetings.

Across programs, however, caregiver perspectives were central from the program's first contact with caregivers. Programs viewed awareness of caregiver perspectives as essential for ongoing, sound, trusting, and reciprocal relationships. Three of the four programs viewed a reduction in caregivers' psychological burden as a nearly immediate benefit resulting from access to respite program personnel who listen, to respect, and learn from caregivers about practical care for their loved ones, and about caregivers' priorities. Program personnel spoke about providing sufficient time and circumstances for caregivers to acclimate and begin developing trust.

Programs offered practical support, including access to resources within and outside of respite services, and made clear from the outset that a responsible and available contact person, and the caregiver-program relationship itself, were among the resources offered.

### Measuring relationships

The *Value of Respite Model* incorporates a life course perspective that begins with the caregiver identifying themselves *as a caregiver*, and recognizes that caregiving contexts vary and change over time. All four programs acknowledged both caregiving and caregiver-respite program relationships as dynamic and contextual, consistent with the *Value of Respite Model*. Each program viewed the relationship as beginning at the first point of contact, and all programs were clear and intentional about the importance of communicating clearly and supportively with caregivers. Program Four emphasized the value of “soft skills”, and the other three programs described the value of attentive listening, the importance of allowing families to move at their own pace and of holding space for that process, and the importance of building trust. Programs also spoke about the importance of offering tangible resources early on and remaining attuned to caregivers' situational and changing need for support.

In another words, according to the programs interviewed, the foundations of strong relationships are established early, with trust and communication viewed as essential to initiating and continuing good relationships. Effective communication is seen as necessary for identifying needed resources, providing anticipatory guidance, making timely service adjustments, and keeping families connected over time. Soft skills are understood as central to relationship building, and may be viewed as clinically sound skills—such as conducting a clinical interview; following up on information gathered during the interview; and offering care that aligns with caregiver identified needs and preferences.

The *Value of Respite Model* assumes some of these soft skills within the concept of person- and family-centeredness. Given the centrality of soft skills and program-caregiver/family relationships described by the programs, however, the *Value of Respite Model* and *Common Data Elements* could be strengthened by including an emphasis on communications that occur between caregivers and programs, and by offering guidance on measures, methods, and processes that capture the extent to which communication occurs regularly, is clear and understood, providers are trusted, caregivers and families feel heard and respected, and resources are well matched needs and are available early on and throughout the duration of respite services.

### Program fidelity: practice implications

**Question 2: *Talk about how you make sure things happen in your program as you have planned*.**

Program fidelity measures are essential in practical evaluation in order to document the relationship between service practice and outcomes. Each of the four programs readily described the processes and checks they put in place to document and monitor services for caregivers and families, and to guide paid and volunteer staff in service provision.

### Practical service monitoring

Program One described a regular, ongoing check-in system in addition to formal caregiver measures.

With our new system, I call about two weeks after a family is matched with a volunteer…Once a month, I am in touch with the volunteers. We touch base and I ask how it's going. Then we do a two-month follow-up, and we do a baseline survey with caregivers. Those are check-in points. I always call caregivers annually for an annual survey. I send regular caregiver resource emails, at least once a month if not a couple times a month. And I may call caregivers with other resources.

For volunteer providers, phone conversations, reference checks, and volunteer responsiveness to the program's outreach helped program leaders gauge the extent to which services and relationships appeared to be working or volunteers needed guidance or support.

We try to have enough touches during the volunteer on-boarding process. Everybody comes with a different circumstance, and lives can change unexpectedly…We create a “no judgement zone” with volunteers. We encourage them to just call us and tell us what is going on. We're not going to be judgy about it… If they have an issue, we’re going to meet them with kindness…We’re going to offer them alternatives that don't make them feel like they've failed. So, all of that goes on sometimes before they are even onboarded…Two times a week we gather together, and we're a small staff, so communication is pretty easy.

The program intentionally demonstrates open communication, trust, respect, and the need to consider changing circumstances in providers' as well as families’ lives.

If (a volunteer) has been with a family for a long time and the person has passed away, they need time to grieve. So, we try to make easy space for them. If they need a break for a few months, we're okay with that.

Program One appears to consider human and relationship processes and fidelity to the model as inseparable. From this perspective, monitoring both volunteer providers' and caregivers' experiences and concerns, along with traditional measures of service dose, for example, could both be seen as essential to service fidelity.

### Continuous person- and family-centered care

Program Two consistently documents all aspects of services and does so in ways that serve the human relationships the program values, and also show that program staff are competent and conscientious about providing care.

We still use carbon copy notes, we’re not computerized. So, our staff still write out everything that happens here. (Our founder) pointed out years ago, and I also think it's important, that it’s such a great communication tool and device to help with that trusting relationship, but also to communicate with their child when they go home—‘Hey, I read that you did this, this, and this. And how was that for you?’

Two keys to program fidelity according to Program Two are individual accountability and overall service consistency.

It's definitely consistency, but it's also individualized. So, every guest has their own file that has their person-centered plan in it, and also has a summary page … that includes what the parent tells me, what the person-centered plan says. And it just really breaks down their complete program for the whole day, including nutrition, using the restroom, how they use it, how often. We train staff to write changes on the paper, and we update the (individualized plan) later. Then there's a paper that the staff sign (acknowledging) that they've read it and are educated and are aware of the changes.

We have constant communication with the families. We call before every single stay to see if there are any medication changes, to see if there are nutritional changes, or overall care changes, and we update those before every visit. Then parents and caregivers know that when they come, they can say, ‘Oh, we forgot to tell you.’ And we fix it on the spot…

The very last thing mentioned in the file is their diagnosis, because we train (staff that) you don't want to treat a child based on their diagnosis. You want to look at the person and what their needs are. Is it important to be familiar with their diagnosis? Absolutely. But that doesn't (tell us) what their needs are. They're so much more than that. Look at the person.

### Staff training and support for service fidelity

In relationship-based respite services, staff training and support are seen as part of delivering services with fidelity. In the following, Program Two talks about the importance of cultivating both a warm social climate and rigorous training to support service delivery.

We have a newer staff, and she stopped in my office yesterday, and she said, ‘I’ve never worked at any place like this before.’ She'd worked at group homes. And the home-away-from-home feeling, when you're at work, and you feel at home. It doesn't quite feel like a job anymore. It seems like a family atmosphere, and enjoyable. She said before she didn't receive much training, and here we provide 90 days and sometimes further than that.

Program Three also views staff training, supervision, guidance, and support as essential for program fidelity.

(New staff) get about 70 h of training before they're ever left alone. By alone, they're never truly alone. They would go into an intake appointment by themselves, but they are always doing a lot of debrief. There is always a program supervisor, not always, but 40 h a week, they are in the same area, available and accessible. We always have (a supervisor) on call for them. Supervisors review all the case notes after visits to make sure all the documentation is accurate, and to identify any red flags or things we need to report but haven't reported. Sometimes it's that case building. When they come in one visit, nothing really stands out. But when they come in 10 or 15 visits over a year, you might (consider), ‘You know, something’s not adding up here. Let’s go in and ask these questions. Let’s offer these services. Let’s see if we can do something more.’

Program Three tailors training to ensure that staff not only understand program expectations but can also communicate clearly and accurately, enabling them to recognize when service adjustments and accommodations are needed over the course of a long-term, dynamic process. The program places value on both program-caregiver relationships and staff-staff relationships, recognizing that strong internal relationships and supportive working conditions directly link to high-quality services for families.

For Program Four, training, supervision, guidance, and support likewise form the foundation of their service model and ensure fidelity to it. In this interview excerpt, a program leader describes how these elements are applied for students and supervisors.

When we get questions from students…it feels like about 90 percent are just things we're helping them navigate—communication, problem solving. And I think they go in thinking that all their learning is going to come from the child and the family, but sometimes in their final journal, in addition to reflecting on that, they talk about how they learned to be flexible and adaptable.

Program Four's leaders continue describing a process that, in addition to encouraging student reflection, is designed to assure that students consider multiple caregiving contexts.

One other thing that really comes together nicely for us is…we have carefully planned these experiences so that (students) are getting a three-pronged approach, and they are seeing children in the three places that children spend most of their time. We have an amazing high fidelity simulation lab, so we have created acute care simulation. And they spend two clinical days in the local schools…in which they often spend time in the disability classroom. And then they spend time at home, and that’s where (the respite program) comes in. So, they get that multi-faceted, holistic approach.

Program leaders from all programs describe respite services as dynamic, relational, collaborative with both families and colleagues, and as reliant on soft skills, all in service of accountability to caregivers and families. Programs emphasized that effective respite is defined not only by *what* activities are delivered, but also by the conditions and qualities that shape *how* those activities are carried out. For researchers and evaluators, the challenge is to determine whether respite results in benefits to caregivers and families, and to identify the specific activities and service conditions that contribute to those beneficial outcomes.

### Considerations for evaluating respite programs and services

Question 3: What advice would you offer to other respite programs considering program evaluation?

### Taking stock of existing evaluation activities

Each of the four programs interviewed brought different evaluation resources and prior experiences that gave them perspective on their current evaluation work.

Program One spoke about the importance of periodically revisiting and revising prior evaluations.

We had an (existing) written evaluation, but it's always a challenge to re-think something. Years ago, our predecessors worked with (a university) school of social work to develop evaluations for all our programs. So, we've been on autopilot. We didn't do a wholesale evaluation of the process. We went back and rethought the whole process, and we changed to adapt, and now we're doing the respite evaluation differently because it’s a different program. It's always a challenge to rethink what you've been doing on autopilot. It was a challenge, but it wasn’t painful.

Program One also spoke about the challenges of collecting and interpreting data in ways that aligned with their theory of change and desired outcomes, and was useful in changing contexts.

Through the project we examined, are we asking the right questions? And some of them were the right questions, but a lot were not. Why do we need to know this? I think it's always a challenge in my work, in social work, some of the work is hard to quantify. How to make things quantifiable. (And recognizing that) a caregiver's scores may go down after a year, and that may be because they've had the burden of another year of caregiving. It doesn't mean that services aren't helping. It's just complicated.

Program One talked about approaches they use to collect and triangulate complicated data and make it more interpretable and useful for program evaluation.

I make notes about whether someone understood a question, for example. And I always include a narrative capturing our conversation… We ask caregivers, what is your overall health rating, and what is your overall stress rating. We talk about that, and talk about how that compares with the previous year. A lot of times I'm not surprised that their health is going down, because they've been caring for a year and maybe not gotten to all their appointments. Or I see the ongoing stress they're going through. I see that a good bit. Their health does go down. It doesn't usually go up. Sometimes, stress can go either way. It sort of depends upon—who knows what their morning has been like. Maybe they've had a terrible day. But I also find that caregivers rate it less. And their adult child will be over here saying, “I think you’re more like a five, Mom.” I think caregivers underrate their stress. They don't want to make it sound like a burden, so they will minimize it.

Program One offered the following advice to respite programs considering evaluation:

If you're not evaluating, you're missing out on a lot of interesting information that will help you improve your program and offer a better service. You're doing families a disservice and yourself by not getting information that will help you be a better service provider and a better partner in their caregiving journey.

### Demystifying evaluation processes

It is notable that Program Two, despite establishing a schedule for strong documentation and monitoring, didn't always recognize these activities as data collection, and perhaps didn't view themselves as evaluators. This may be evidence that for respite programs, evaluation jargon needs to be reduced and evaluation processes demystified. The following conversation that occurred near the end of an interview points to program activities that are essential to performance measurement but not necessarily viewed by programs as evaluation.

**Interviewer:** As I hear you talk, and this has been true of all the programs, you already do so much evaluation, but you don't think of it as evaluation. I think part of it is fitting it into a framework in your mind. So, when you do the intake, you have a really clear process.
**Program Two:** Yes. To the point where I have it all typed out. We give it to the family, so that they know this is how things (will occur).
**Interviewer:** You have this laid out, you have a training curriculum, you have supervision for 90 days. You definitely have a curriculum map, and you're monitoring it…One of the biggest things you are doing is the individual plans. That is evaluation. You are saying what is important to know and do, and you are checking to see if you have done it. The third piece is, is there a good outcome. Did they actually get the care that you intended? And when that doesn’t happen, you have a way of checking that out. You are doing continuous quality improvement.
**Program Two:** And incident reports. The state requires (monitoring of medication, wrong medication, schedule not followed). That really helps us. I try to reiterate to staff it’s not a punishment thing, it’s accountability… If we're having a lot of missed meds, why is that happening? What do we need to change in our med sheets or education to make sure that isn't happening?

Program Two's leader shared that, “I shy away from asking for help, and I think I have to figure it out myself.” The *Value of Respite Model* and *Common Data Elements* offer a usable framework for program evaluation that may help demystify measurement processes and provide structure in designing and conducting program evaluation. But every one of the four programs we worked with for the evaluation project also needed additional hands-on support and a process for thinking aloud with colleagues about their program goals and theory of change, and for directly linking their deep knowledge about caregivers and families and the services they offer to evidence of the benefits of respite services.

### An evaluation framework that accommodates program phases, priorities, and practices

Program Three reinforces the notion of a straightforward framework, one that starts simply but that also can accommodate increased complexity, and guidance and support in using it. When asked to offer wise advice to other programs wishing to evaluate respite services, Program Three offered this:

Do it. Start simple. Seek help. I’m hopeful that what we learned from this, other people can use too. It should be more of not should we, but how do we. And I think, again, just start simple, and layer on top of it. And like the conversations with you, I never really thought about this as data that we're collecting, and what do we do with it. But asking for help. Who are the experts out there that can help? Capitalizing on that. One of the things I loved about this cohort is you give some education, but we also get to talk through the real life-ness of it. It's not just a theory or idea, but also the practicality of it.

Even with a straightforward framework for evaluation, conducting an evaluation requires thinking in complex and nuanced ways. Program Three, like all the programs interviewed, made astute observations about data collection methods and how the data they collect may be used to interpret and understand outcomes.

This is one of the things we've added (to data collection). We have parents self-identify their reason for use. When they call and say I have a doctor's appointment, we check ‘doctor’s appointment’ on the form, and that's the only thing we check. But we know that there's so much more going on with that family. So, we're going to add in primary reason and secondary reason. For secondary reason, our staff will check boxes for all the reasons that our staff knows, like high levels of stress, or poverty, or single parenting, or domestic violence, so that we have a better picture. These are the things our parents don't want to lead with, but if we know they're going on, that just because you're going to a doctor’s appointment, it doesn't mean all those other things aren't there.

Program Four provides insights into capturing aspects of care provider––caregiver/family relationships that develop over time and appear, based on our small sample of Innovative and Exemplary Respite Services programs, to be essential to achieving beneficial respite outcomes.

I can see a lot of growth and learning happening with the students. For example, last semester we had (a very young child) in our program, and the students assigned to the family felt like babysitters the whole time, which in some sense they were. Throughout the reflection they started talking about the family, and how this affected the family. This was the (family's) third child, and you could slowly see the gears turning in (the students’) brains, the reframing and how they talked about the experience was completely different too.

This passage illustrates the importance of student providers' beliefs and attitudes toward the families they work with, as well as the nature of the work and the importance of trainers' and supervisors' awareness of these perspectives. This awareness is necessary to inform supervisors' guidance to volunteers and staff in learning to listen to, consider, incorporate, and respond to caregivers' and families' perspectives when designing, delivering, and adapting respite care.

## Discussion

The *Value of Respite Model* and companion document *Recommended Common Data Elements* were developed expressly for the purpose of advancing respite research. The authors of these documents note that “common data elements may also be useful for respite program evaluation,” to evaluate program efficacy over time and allow comparison to other service models (2).

Based upon what we learned from the four respite programs using the *Value of Respite Model* and the *Recommended Common Data Elements* to plan and conduct practical evaluation, did the Model serve as a suitable and useful framework for evaluation? And were the *Recommended Common Data Elements* helpful to programs in selecting measurement processes and tools that aligned with the model?

The *Value of Respite Model* is based on a human or social-ecology model, a Life Course Framework, and Family Self-Management Theory. The Model includes three elements:

1. **Context**, focusing on caregiver-care recipient relationships within family, community, and public policy contexts that help protect caregivers or put them at risk for negative outcomes;
2. **Process**, focusing on a caregiver’s identification as a caregiver needing and accepting respite, access to appropriate services, and achieving goals with satisfactory outcomes; and
3. **Outcomes**, focusing on the value of respite reflected in improved health and wellbeing in physical, mental, social, spiritual, and/or financial domains and improved quality of life for individuals and the family as a whole.

The Model specifies that relationships are neither one-directional nor linear, and that context changes over time and across the life course.

The Model was well-suited for evaluation in that every program expressly considered the caregiving Context for individual caregivers and families when designing and delivering respite services. The Process element of the Model emphasizes including caregivers and families as partners in shaping services that suit their unique circumstances and needs, and all four programs intentionally included caregiver voices from the first point of contact. This inclusion was accomplished through the development of relationships between caregivers, families, and respite programs, supported by strong communication that allowed for timely monitoring and adaptations of respite services to address caregivers' and families' changing circumstances and priorities.

Where the Model and common data elements perhaps fall short for the purposes of program evaluation is in the absence of specific measurement tools and processes that capture the development of trust and strong communication between caregivers and programs over time—relationships that guide service delivery and form the foundation for subsequent outcomes. This limitation lies not only in the Model itself, but in the respite field broadly. The soft skills that programs see as hallmarks of their work are measurable skills, but we are just beginning to understand when and how to measure them. A broader survey of respite programs perspectives and observations regarding the importance of measuring soft skills like communication and trust could help determine whether respite providers more generally share the views expressed by the four Innovative and Exemplary Respite Programs. For programs that prioritize soft skills, relevant common data elements and measurement tools and processes need to be identified and incorporated into the *Value of Respite Model*.

A second area that may be problematic with using the Outcomes element of the Model for program evaluation is that outcomes identified in the common data elements, while comprehensive and grounded in common-sense, originate primarily from researchers rather than from caregivers and families themselves. The concept of “well-being” has typically been assessed in respite research and evaluation through the use of measures—of stress or caregiving burden, for example—designed for other service settings. These measures may not reflect individual caregivers' and families' ideas about what constitutes wellbeing in the context of respite. During monthly evaluation seminars, the four programs spoke frequently about many of the data elements listed in the *Value of Respite Model* including global health, fatigue, stress, loneliness, and quality of life. For programs, however, critical questions remain: how to measure these accurately, unobtrusively, and without undue burden on caregivers; when to measure them given changing caregiver contexts; and whether these outcomes reflect what individual caregivers themselves consider most important. The interpersonal and clinical skills needed by program staff to develop strong, enduring caregiver-program relationships are more nuanced and challenging to measure, yet they may be among the most important qualities of respite to understand, support, and encourage, along with and in relation to measures of caregiver wellbeing.

Model fidelity was a strength of all four programs and seemed to be valued on par with pre- and post-measures of constructs such as caregiver stress or burden. In relationship-based service models, measuring fidelity is an ongoing process that requires vigilance, reflection, and especially, confirmation from caregivers and families that they are receiving what was promised and that those services are working as anticipated. Programs appear to understand this intuitively, and it is part of their ethos of serving families.

This underscores the need to support respite programs wishing to incorporate service fidelity monitoring into practical evaluation. The four programs point the way forward through their descriptions of intake and review processes, service documentation and monitoring practices, staff training and communication strategies, and most notably, relationship building and ongoing monitoring. The *Value of Respite Model* and especially the Common Data Elements Process measures, *Description of Respite Model Tool* and *Caregiver Experience with Respite Tool* (9), show promise for service documentation and monitoring.

An overall strength of the *Value of Respite Model* for practical evaluation is its comprehensive and coherent inclusion of contexts, processes, and outcomes. The Model enables program evaluators to identify mediating contextual variables; to support documentation of service processes that may influence outcomes; to promote data triangulation; and to encourage hypotheses generation—an especially important function during early phases of program development.

Program evaluation is a discipline loaded with jargon and concepts that can make it opaque, confusing, intimidating, and overwhelming to many respite program providers. To programs skilled in person- and family-centered services that prioritize program-family relationships nurtured over time, the language of summative vs. formative vs. process evaluation, categorical vs. ordinal vs. interval data, or positivism vs. interpretivism vs. constructivism approaches can seem foreign, indecipherable, and of little relevance to the day-to-day work of designing and delivering respite services. That is not to say these concepts and terms are irrelevant or unimportant. Rather, in my view, they are not where most programs should begin.

The knowledge and insights programs shared, combined with the use of the *Value of Respite Model* and *Common Data Elements* as organizing and instructional structures, informed ARCH's thinking about the Model's utility for practical evaluation. As one program leader mused, although her program had long collected data, the purposes and uses of data collection were not always clear. “Now,” she stated, “It is intentional.” Following completion of the project, all four programs continue to use their evaluation plans in day-to-day program practice, three have developed data briefs documenting the merit of their work, and one university-based program has submitted a proposal to their Internal Review Board to expand its initial evaluation work.

We conclude that most programs need an organizing structure and basic template to introduce and guide entry-level program evaluation, and that the *Value of Respite Model* structure and measurement template can serve as a strong foundation for conducting or strengthening program evaluations. The structure of the *Value of Respite Model* also may help to “bound”, or limit the focus of, an evaluation, making it manageable for respite programs, thereby encouraging and empowering them to engage in evaluation activities.

We offer the following recommendations for the development of a template that capitalizes on the *Value of Respite Model* and Common Data Elements.

1. In addition to the three data sources in the *Value of Respite Model* (Context, Process, and Outcomes), the template should be grounded in a program’s unique purpose and goals; goals should be the starting point for a “theory of change” so that the evaluation is logical, and goals should guide the selection of outcome measures.
2. We recommend that in addition to Caregiver and Care Recipient Contexts, life circumstances data should be collected periodically. Programs tell us that life circumstance data informs service development and adaptations, and it helps programs interpret outcomes.
3. In terms of Respite Process, every program unequivocally explained in their interviews that “building and nurturing trusting relationships with caregivers” was essential and fundamental, and that these foundational relationships begin during intake and take time and effort to grow. Available measures fail to adequately capture signifiers of strong relationships such as trust and communication, and do not describe whether or how programs foster and maintain positive relationships with families.

In summary, the *Value of Respite Model* provided a logical and coherent model that was valuable in teaching and supporting respite programs as they designed and conducted practical program evaluation. The Model helped to demystify evaluation and bridge a gap between evaluation as a professional discipline, and evaluation as a practice fundamental to programs wishing to “get things right” for caregivers and families, and to demonstrate the value of their work (14).

## Data Availability

The datasets presented in this article are not readily available because raw qualitative data are not available for sharing. Requests to access the datasets should be directed to Susan Summers, sjsummers@archrespite.org.
